# Smartphone-Based Self-Monitoring in First Episode Psychosis: Mixed-Methods Study of Barriers and Facilitators to Engagement

**DOI:** 10.2196/71989

**Published:** 2025-08-26

**Authors:** Maria Chiara Del Piccolo, Ryan Hammoud, Maisie Khan, Vanessa Shanon D'Costa, Aljawharah Almuqrin, Anna Georgiades, Stefania Tognin, Andrea Mechelli

**Affiliations:** 1 Department of Psychosis Studies, Institute of Psychiatry, Psychology & Neuroscience, King's College London, De Crespigny Park, London, SE5 8AF, United Kingdom, 44 (0)20 7848 0289; 2 Department of Health Sciences, College of Health and Rehabilitation Sciences, Princess Nourah bint Abdulrahman University, Riyadh, Saudi Arabia; 3 Brent Early Intervention Service, Central and North West London NHS Foundation Trust, London, United Kingdom

**Keywords:** mHealth, psychotic disorder, schizophrenia, digital technologies, relapse monitoring

## Abstract

**Background:**

Despite growing interest in smartphone technologies to monitor patient outcomes, only a limited number of studies have investigated the long-term use of smartphone applications in patients with psychosis. Therefore, we have a limited understanding of facilitators and barriers to sustained engagement with these technologies in this population.

**Objective:**

This study aimed to examine the impacts of demographic and clinical characteristics on engagement with smartphone-based assessments in people who experienced a first episode of psychosis (FEP); to assess acceptability, barriers, and motivators influencing long-term engagement; to understand how to improve users’ experience in future smartphone-based research; and to better understand participants’ attitude toward the use of digital technologies in clinical settings.

**Methods:**

This study used a mixed-methods design. Linear regression models were used to assess the association between demographics and clinical variables (global assessment of functioning [GAF], global functioning [GF], Positive and Negative Syndrome Scale [PANSS], and Hamilton Rating Scale for depression [HAM-D]) and the proportion of completed app-based assessments during the 12-month period. Qualitative data were analyzed using a hybrid deductive and inductive thematic approach.

**Results:**

A total of 274 participants were recruited. The average smartphone-assessment completion rate was 14.5% (53/365 daily assessments completed over a 1-year period). No statistically significant associations were found with demographic characteristics, while there was a positive association with the baseline level of functioning (GAF disability: coefficient 0.173; 95% CI 0.022-0.324), GAF symptoms (coefficient 0.155, 95% CI 0.011-0.298), and GF social (coefficient 2.27, 95% CI 0.474-4.061). In contrast, the completion rate was negatively associated with PANSS general subscales (coefficient, −0.589, 95% CI −0.923 to −0.254) and depression (coefficient, −0.524, 95% CI −0.874 to −0.174). A total of 20 participants completed the qualitative sub-study. Participants acknowledged the ease of using the app and the user-friendly interface. They also valued the repetitive nature of the assessments, noting that it encouraged self-reflection. However, daily assessments felt time-consuming and repetitive over time, and some participants found it challenging to remain engaged due to competing life priorities. While some participants felt the app lacked tangible benefits, others reported indirect benefits from tracking their daily experiences. The opportunity to contribute to research was highly valued, as well as the importance of human connection with the researchers in sustaining long-term engagement. Participants made several suggestions: reducing the frequency of smartphone assessments to 1‐2 times per week, integrating passive and active monitoring tools, and incorporating individualized features. Most participants supported the idea of using an app as part of their clinical care, provided data security and privacy were adequately addressed.

**Conclusions:**

Our study suggests that smartphone-based technologies are an acceptable monitoring tool for patients with first episode of psychosis. While participants expressed interest in using smartphone apps as a clinical tool, they emphasized that this should complement, rather than replace, the therapeutic relationship. Patients experiencing more severe symptoms may require additional support and incentives to sustain engagement over time.

## Introduction

### Background: The Promise of Digital Psychiatry

Psychosis, which directly affects 2%‐3% of the population, is a severely debilitating psychiatric disorder characterized by a range of symptoms, including abnormal perceptions and false beliefs, negative symptoms, and cognitive deficits [1]. Despite the efficacy of antipsychotic medications, up to 80% of patients with the first episode of psychosis (FEP) will relapse within 5 years of remission from the initial episode [1,2]. Prediction and prevention of relapse remain key clinical targets in psychosis management; however, traditional assessments have been criticized for being lengthy, inconsistent, and prone to recall bias [3,4].

Given the increasing ubiquity and flexibility of smartphone technologies [5,6], mobile apps have emerged as a promising tool for use in patients with serious mental illness [3,4,7-9]. Specifically, smartphone apps allow the close monitoring of people across multiple contexts, time points, and locations. In research settings, the use of smartphone technologies offers high fidelity, as all participants use the same tool independently, without the involvement of an intermediary researcher. These tools also provide access to a wealth of information through both active and passive data collection (such as step count and distance walked) via background tracking [10]. In clinical settings, smartphone apps could serve as valuable tools for monitoring and predicting patient outcomes. However, despite increasing interest in digital psychiatry, the field has been criticized for overlooking the importance of tailoring these technologies to users’ needs [11] and concerns remain about long-term user engagement and real-world implementation [12,13]. Furthermore, while adherence to the use of smartphone tools seems to be acceptable [11,13], long-term user engagement appears to be influenced by several factors, including demographic characteristics, mental health symptoms, perceived benefit, and level of support provided [14]. As the impacts of these factors are not well understood, data from long-term naturalistic studies are needed to better understand facilitators and barriers to user engagement in specific clinical populations [15] and facilitate the adoption of these technologies into the health care system [11,16]. Despite evidence of lower engagement in individuals with a psychotic disorder [17], few qualitative studies have investigated the acceptability of long-term smartphone apps for monitoring and predicting symptoms in patients with psychosis [7], leaving a gap in our understanding of motivation and barriers in this clinical population.

### Present Study

To address this gap, we conducted a mixed-method study [18] in a large sample of individuals with FEP. By integrating quantitative and qualitative data, we aimed to better understand patients’ experiences, motivations, and challenges in the long-term use of smartphone apps for self-monitoring. We hope that this information can be used to improve engagement with digital technologies in future studies. The specific objectives of this study were as follows: (1) to examine the impacts of demographic and baseline clinical characteristics on completion rates of smartphone-based assessments; (2) to assess acceptability and to identify barriers and motivators that may influence long-term user engagement with the study app; (3) to understand how to improve users’ experience and engagement in future smartphone-based research in FEP; and (4) to better understand participants’ general attitude toward the use of digital technologies in clinical care settings.

## Methods

### Overview

This study was nested within the *Social Environment and Early Psychosis*. Using a prospective longitudinal design, the main study was designed to develop and validate a smartphone app (Social Mind app) to detect some early warning signs, particularly daily social stress, considered significant to predict the risk of relapse in patients with psychosis at the individual level [19-21]. For 12 months, the Social Mind app sent participants prompts to complete daily smartphone-based short assessments focused on social interactions and affect, alongside passive data collection (eg, distance traveled and location). In addition, clinical data were collected directly via structured clinical interviews at 4 time points and indirectly via access to electronic health records.

### Ethical Considerations

Ethical approval was obtained from the London-Surrey Research Ethics Committee (20/LO/0331). All participants provided written informed consent and were informed that they could withdraw from the study at any time without providing a reason and without any impact on their medical care or legal rights. Each participant was invited to attend a total of 4 study appointments to complete the assessments and was compensated £40 (equivalent to US $ 53.65) each time, either via bank transfer or voucher, based on their preference. All the data collected were de-identified using numerical codes and securely stored in accordance with GDPR guidelines.

### Study Design

This study used a convergent mixed-methods design where quantitative and qualitative data were collected and analyzed concurrently, then integrated through narrative at the interpretation and reporting level [18].

### Quantitative Data

#### Participants

Participants (n=274) were recruited between December 2020 and December 2023 from 10 Early Intervention Services for Psychosis in England and Wales. Participants were initially identified through convenience sampling and selected through purposive sampling either by clinicians at the Early Intervention Services for Psychosis or researchers at each site. Participants were eligible if they were aged 18‐40 and had experienced a FEP in the 24 months prior to entering the study as defined by a *DSM-IV* diagnosis of schizophrenia, schizophreniform disorder, schizoaffective disorder, or delusional disorder based on the Structured Clinical Interview for DSM-IV (SCID-I) [22]. Finally, participants were required to have access to a smartphone with internet data. Participants were excluded if they experienced more than one previous FEP; if they refused to complete clinical interviews and smartphone short assessments; if they were unable to provide informed consent; if they had an estimated IQ<70; or if they were sectioned under the Mental Health Act 1983.

#### Data Collection and Procedures

Demographic and clinical data were collected during study assessments often conducted online by video call at baseline, and at 4-, 8-, and 12-month follow-up. For the purposes of this paper, we will only consider data collected at baseline. Participants’ current psychotic symptoms were assessed using the Positive and Negative Syndrome Scale (PANSS) [23]; presence and severity of depressive symptoms were assessed using the Hamilton Rating Scale for Depression (HAM-D) [24]; and level of functioning was assessed using the Global Assessment of Functioning (GAF) symptoms and disability [25] and Global Functioning (GF) role and social scale [26].

After the baseline, participants were invited to download the Social Mind app on their phone and briefed on its usage. During the following 12 months, participants received a daily prompt and were instructed to complete as many assessments as possible, at least twice a week. The daily assessments lasted approximately 2‐3 minutes and consisted of questions on early warning signs, including the individual’s previous night’s sleep (eg, Did you have trouble falling asleep last night?), momentary social contact (eg, Who is with you right now?), social contact over the past 24 hours (eg, Who have you been with in the past 24 h?), momentary social stress sensitivity (eg, I find being with them stressful), and social stress sensitivity over the past 24 hours (eg, I found it relaxing to be alone). In addition, information about the exact geographical location of each assessment and background location was acquired using geotagging location and information regarding physical activity (step count, distance walked/cycled/ran). The Social Mind app was developed using Citizen Scientist [27], a platform that allows researchers to create customized smartphone apps for data collection.

#### Statistical Analysis

Linear regression models were used to assess the association between demographics and clinical variables and completion rates. The dependent variable in all models was the completion rate, defined as the proportion of completed app-based assessments during the 12-month study period. Separate models were run for each independent variable of interest (demographic characteristics, level of functioning, depression, and psychotic symptoms at baseline). All models were then adjusted for age, gender, ethnicity, employment, and education status. Stepwise regression techniques were not used; variables were entered into models based on a priori theoretical justification. Findings were considered significant when *P*<.05.

Finally, to assess whether the participants interviewed were representative of the full sample, we conducted between-group comparisons. The normal distribution was assessed using the Shapiro-Wilk test of normality. The Fisher exact test was used to analyze categorical data. Continuous data were analyzed using independent 2-tailed t-tests or the Wilcoxon rank-sum test for non-normally distributed data (significance *P<*.05). Data were analyzed using STATA MP (version 18) [28].

### Qualitative Data

#### Inclusion Criteria and Recruitment

Participants were recruited by email or phone from South London and Maudsley and Central and North West London NHS Foundation Trusts, the 2 main recruiting sites for the Social Mind main study. Specifically, we recruited individuals who had either completed the 12-month period or had been using the app for a minimum of 8 months to ensure they had sufficient time to test the app. We adopted a purposive sampling strategy to maximize variation in order to gain insights into a wide range of experiences and perspectives. A total of 41 participants were identified and triangulated based on completion rates, gender, and ethnicity. A follow-up email or phone call was made to non-responders. Recruitment ended when data saturation was reached.

#### Data Collection and Procedures

Semi-structured interviews were conducted between December 2023 and September 2024 by MK and VD (Research Assistants and MSc students) and MCDP (Research Assistants and PhD student) with previous experience of qualitative research. Interviewers had no prior relationships with participants, but they had occasionally met in the context of the main research study (eg, occasionally the interviewer also carried out the follow-up study assessments). Interviews were guided by a pre-established topic guide designed by the Social Mind research team and were conducted and recorded online through Microsoft Teams. The focus of the interview was to explore the experience of participants taking part in the study, covering the app as well as their general views on the implementation of mobile apps in clinical care settings (the topic guide is available in Multimedia Appendix 1). Only participants and researchers were present during the interviews.

#### Analysis

Following the interviews, recordings were transcribed verbatim, pseudo-anonymized, and imported into NVivo 14 for analysis. For the analysis, we used a concept-driven 6-stage hybrid approach of deductive and inductive coding and a thematic approach [29]. This involved the following steps: (1) a codebook was developed a priori based on the research questions (the topic guide) and previous literature [11,14,16,30]; (2) the codebook was tested for reliability via double coding and coding comparison; (3) we then summarized data and identified preliminary themes; (4) the revised codebook was applied to the remaining transcriptions; (5) final themes were identified; and (6) in the interpretative phase, themes were further clustered and described.

#### Methodological Orientation and Reflexivity

This research was conducted in a critical realist paradigm, accepting that scientific observations (qualitative data ie, interviews) are part of a multi-dimensional system and are shaped by the conceptual frameworks within which scientists and participants operate, therefore acknowledging the role of social context [31]. MCDP is a PhD researcher investigating the effects of social stress in patients with FEP with experience of working with individuals with severe mental health conditions both in clinical and research settings; at the time of the analysis, MK and VD were MSc students in the Early Intervention in Psychosis MSc (King’s College London) with prior experience of working with patients or study participants—they both received internal training and regular supervisions from the other members of the research team; RH (PhD) is involved in several studies investigating digital health technologies and was involved in the designing and maintenance of the study app—he was primarily involved in the quantitative analysis; AM (Professor of Early Intervention in Mental Health, clinical psychologist, and Chief Investigator for this study) has vast experience devising and conducting studies focusing on early intervention in mental health, digital health technologies, and impact on environment on mental well-being. Although we opted for a primarily deductive ‘close to the data’ approach to analysis, the researchers might have brought certain expectations, particularly about the study app and its likability and acceptability. To minimize potential biases, the topic guide was designed with neutral, open-ended questions, and all interviews were conducted by researchers who were not involved in the study design or app development and therefore had limited attachment to the app. Furthermore, MK and VD independently coded the data and had regular meetings for coding comparison. Finally, MCDP re-coded all data using the revised codebook. Reporting adheres to guidelines for qualitative research (Consolidated Criteria for Reporting Qualitative Research; COREQ) [32], which are available in the supplementary materials in Multimedia Appendix 2.

## Results

### Sample Characteristics

A total of 274 participants were recruited in the Social Mind main study. Of these, 20 completed the qualitative sub-study. Sociodemographic characteristics and completion rates are reported in Table 1.

**Table 1.** Between-groups comparisons (interviewed vs not interviewed) for completion rate and baseline characteristics.

	Full sample (n=269)	Interviewed (n=20)	Not interviewed (n=249)	*P* value
Completion rate, mean (SD)	14.52 (16.08)	22.37 (25.25)	13.85 (14.92)	.058
Age, years, mean (SD)	25.6 (5.3)	26.1 (4.3)	25.6 (5.4)	.385
Gender, n (%)				.352
Male	151 (56.1)	9 (45.0)	142 (57.03)	
Female	118 (43.9)	11 (55.0)	107 (42.99)	
Ethnicity, n (%)				.598
White	99 (36.8)	6 (30.0)	93 (37.4)	
Black	75 (27.9)	8 (40.0)	67 (26.9)	
Asian	45 (16.7)	2 (10.0)	43 (17.3)	
Other	50 (18.6)	4 (20.0)	46 (18.5)	
Employment,^a^ n (%)				.494
Employed	136 (52.1)	12 (60.0)	124 (51.5)	
Unemployed	125 (47.9)	8 (40.0)	117 (48.5)	
Education,^a^ n (%)				.796
In education	73 (27.2)	6 (30.0)	67 (27.0)	
Not in education	195 (72.8)	14 (70.0)	181 (72.9)	
Positive and Negative Syndrome Scale (PANSS), mean (SD)				
PANSS positive	9.07 (3.04)	8.45 (2.39)	9.12 (3.08)	.401
PANSS negative	10.57 (3.67)	9.2 (2.74)	10.68 (3.72)	.059
PANSS general	23.59 (6.49)	20.8 (4.86)	23.83 (6.56)	.027^b^
PANSS total	43.11 (10.95)	38.45 (7.53)	43.53 (11.12)	.039^b^
Hamilton Rating Scale for depression (HAM-D) score, mean (SD)	6.24 (6.03)	5.15 (6.09)	6.33 (6.03)	.321
Global functioning (GF) role, mean (SD)	7.32 (1.40)	7.8 (1.47)	7.28 (1.39)	.038^b^
GF social score, mean (SD)	7.54 (1.23)	8.15 (.99)	7.49 (1.24)	.031^b^
Global assessment of functioning (GAF) disability score, mean (SD)	71.79 (14.51)	77.5 (14.08)	71.32 (14.47)	.041^b^
GAF symptom score, mean (SD)	71.66 (14.65)	76.5 (15.13)	71.2 (14.57)	.196

a Due to missing data, numbers may not add up to the total.

b A statistically significant difference was found between interviewed and not interviewed participants.

### Quantitative Analysis

**Figure 1.**
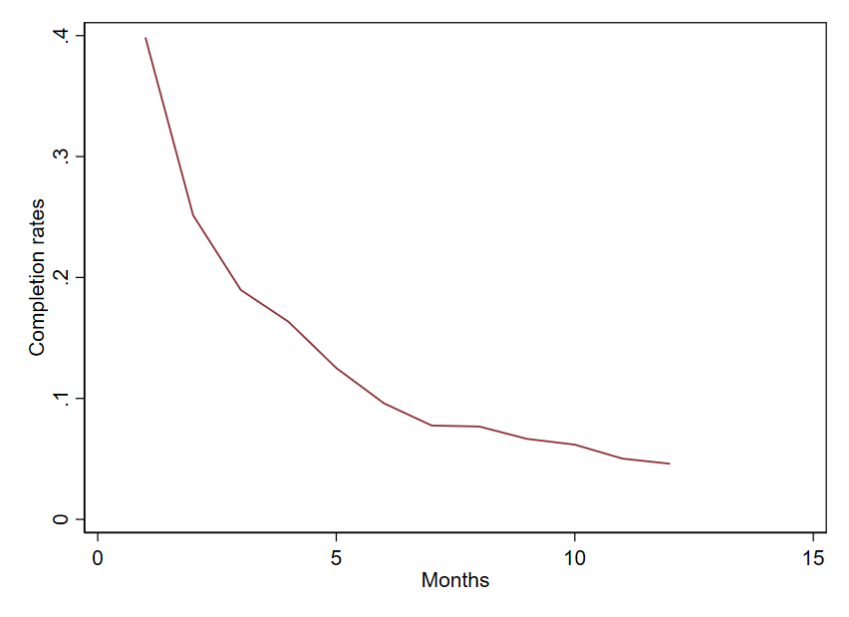
Average monthly completion rate over the 12-month study period.

The average completion rate for the smartphone-based assessment was 14.5% (SD 15.96), with a linear trend showing a decrease in monthly compliance over the 12 months (Figure 1).

### Association Between Demographic Characteristics and Completion Rates

Simple linear regression models indicated no statistically significant associations between demographic characteristics (age, gender, ethnicity, employment, and education status) and smartphone-based assessment completion rates (Table S1 in Multimedia Appendix 3).

### Association Between Baseline Level of Functioning and Completion Rates

**Figure 2.**
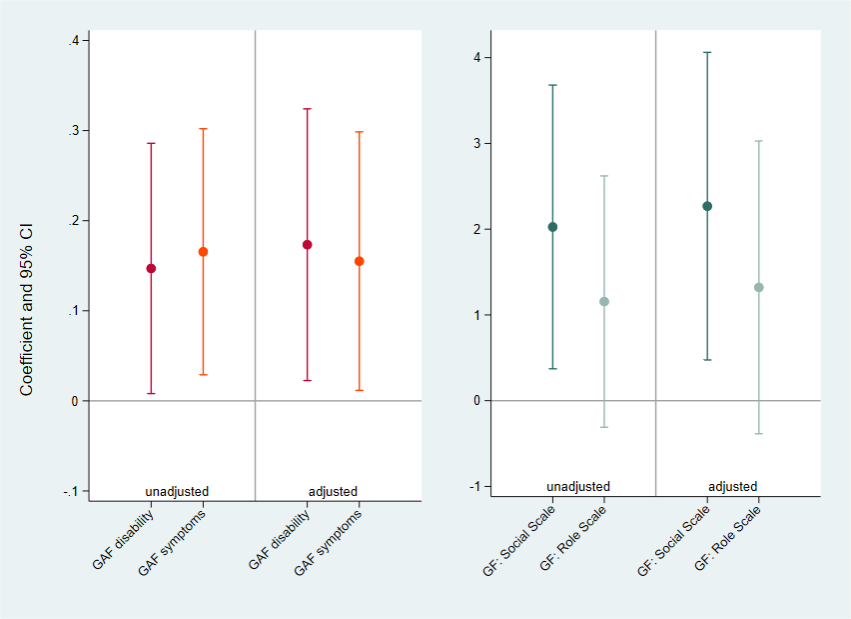
Association between baseline level of functioning and smartphone-based assessment completion rates. GAF: global assessment of functioning; GF: global functioning.

A positive association was found between baseline level of functioning and smartphone-based assessment completion rates: specifically, this was evident for baseline GAF disability score (coefficient 0.173, 95% CI 0.022-0.324), GAF symptoms score (coefficient 0.155, 95% CI 0.011-0.298) and GF social scale (coefficient 2.27, 95% CI 0.474-4.061). No statistically significant association was found for the GF role scale. These results were confirmed after adjusting for potential confounders, as shown in Figure 2.

### Association Between Symptoms of Psychosis at Baseline and Completion Rates

**Figure 3.**
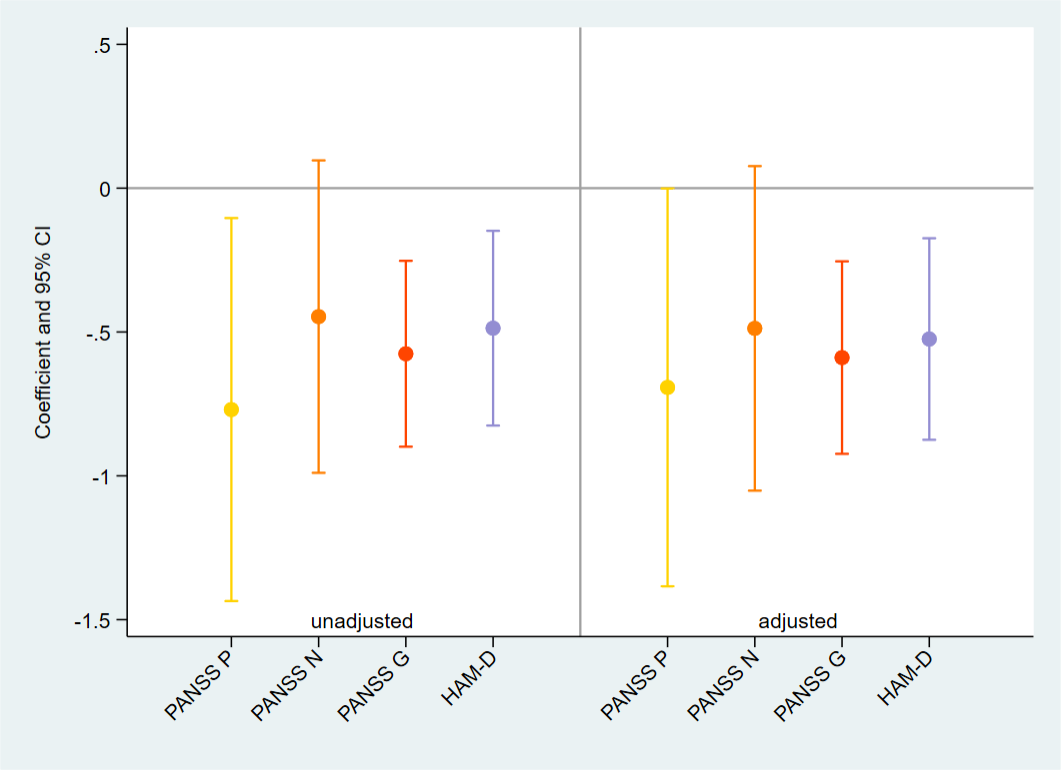
Association between baseline clinical characteristics and smartphone-based assessment completion rates. PANSS: Positive and Negative Syndrome Scale. P: positive scale; N: negative scale; G: general psychopathology scale; HAM-D: Hamilton Rating Scale for Depression.

Smartphone-based assessment completion rates were negatively associated with baseline PANSS general symptoms scores. This association remained statistically significant after adjusting for potential confounders (coefficient −0.589, 95% CI −0.923 to −.254). Unadjusted PANSS positive symptoms were also negatively associated with completion rates, but this association was no longer statistically significant after adjustment, as shown in Figure 3. No statistically significant association was found between completion rates and negative symptoms.

### Association Between Symptoms of Depression at Baseline and Completion Rates

Smartphone-based assessment completion rates were negatively associated with baseline severity of depressive symptoms measured using the HAM-D (coefficient −0.524, 95% CI −0.874 to −.174). These results were confirmed after adjusting for potential confounders, as shown in Figure 3.

### Qualitative Analysis

We conducted 20 semi-structured interviews lasting between 12 and 21 minutes (refer to Table 1 for demographic characteristics). At the time of the interview, all participants had completed at least 8 months in the study and were triangulated based on completion rates, gender, and ethnicity to maximize sample representativeness. The final codebook is reported in Textbox 1.

Textbox 1. **Codebook used for the qualitative analysis.**

**Deductive**
  - App features
    - Positive sentiment: User-friendly, repetitive nature and structure of the assessments.
    - Negative sentiment: Poor integration into daily life, lack of clarity (daily assessments and ratings), and questions about relevance, frequency, and repetition.
  - Motivation
    - Internal: Contributing to research, perceived benefits on mental health
    - External: Money incentive, good experience with researchers, notifications
  - Barriers
    - Technical difficulties, reminder or notification, lack of personal benefit, lack of technical app support, other life priorities
**Inductive**
  - App improvements and suggestions
    - Lowering frequency and shortening length of smartphone-based assessments, app visual appeal, features including gamification, peer support and community, question variety
  - Attitudes towards apps in clinical care
    - Negative attitudes
    - Positive attitudes
    - Unsure, indifferent, and mixed attitudes
  - Data ownership
    - Privacy, confidentiality, and data ownership

#### Acceptability

**Figure 4.**
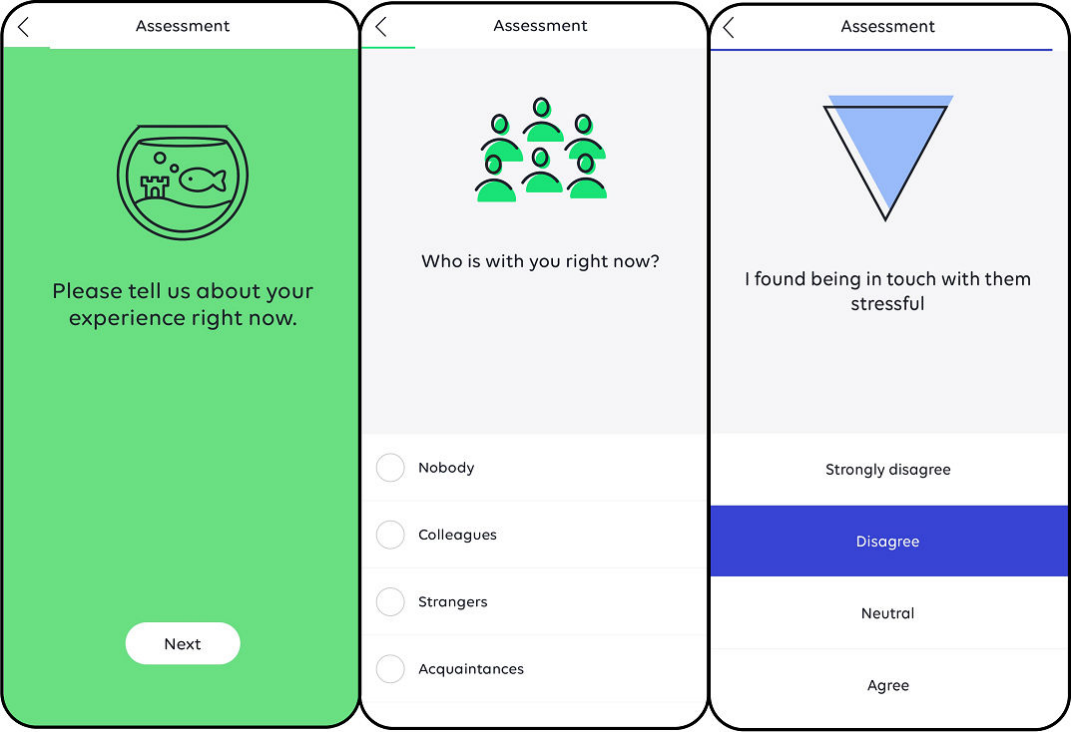
Screenshot examples from the Social Mind study app.

Most participants found the app easy to download and set up or praised the researchers for their supportive guidance during this process. Initial impressions of the app were also positive: participants found the design and interface user-friendly (see Figure 4) and the app straightforward to use.

Notifications, prompting participants to complete the assessments at the scheduled time, played a key role in maintaining engagement. Participants valued the consistency of the assessments, which fostered familiarity with the questions and made the process more efficient over time.

I had just come out of being in hospital for me regiment and like structure like was really important to me, so I was trying to keep with like things that like give me structure.

[Participant 16, 15% completion rate]

Only a few participants enjoyed completing the smartphone-based assessments daily, while the majority felt that once or twice a week would have been enough. Despite individual assessments being brief (2-3 minutes), participants reported that over time they felt time-consuming and repetitive. Due to the daily frequency of the assessments, some participants felt they had not had enough time to gauge their emotions and mood between assessments, making it harder to answer some of the questions.

I didn' t like that we had to use it every day. It became very tedious very quickly, especially because like the…. the questions and answers- Especially my answers would be the same about nearly every day.

[Participant 18, 21 % completion rate]

#### Motivators to Sustained Engagement

A key motivator for participants, both for joining the study in the first place and for maintaining their engagement, was the opportunity to contribute to research. They valued the chance to help advance our understanding of psychosis, support others who may develop the illness in the future, and give back for the support they had received.

I think just helping to contribute to research for psychosis, helping people […] the age group of young people to prevent relapse was something that I actually cared about. That was the only thing that really drove me most days […].

[Participant 14, 32% completion rate]

Despite the fact that the Social Mind app is a monitoring app, and therefore was not designed to provide mental health support, participants reported that it helped them with self-monitoring and self-reflection.

At the time I was going through…umm…clinical depression and I was a bit isolated a lot as well (be)cause… I had just moved out so I thought the app would help in terms of just, you know, it’s almost like a diary sense. Like you just kept checking in on yourself every day.

[Participant 4, 11% completion rate]

The relationship with researchers further enhanced engagement and contributed to overall positive appraisal of participation. Many participants appreciated the 4-monthly assessments with a researcher, which were seen as more useful and enjoyable than using the app. Specifically, these sessions provided a dedicated time to reflect on the previous months and discuss not only their symptoms but also broader aspects of their lives.

…the catch-ups […] were very… always very supportive because we discussed a lot of how I was feeling and I felt that was a very special time of my life, that I was very fragile. So, I felt better.

[Participant 19, 17% completion rate]

#### Barriers to Sustained Engagement

Although very infrequent, some participants expressed confusion about using the app’s Likert scales, reporting that the options could be *ambiguous* to understand. Generally, participants did not experience technical issues with the app, but some reported not receiving the daily notification or the app performance being slow. A lack of technical support was also mentioned: some participants reported technical difficulties with the app but did not receive support, even after reporting the issue via the app. Others did not know how or where to report technical issues. These negative experiences affected app usage and completion rates:

I was doing it like daily and then it… I got annoyed with the slowness and then I kind of stopped doing it and I kind of hardly ever did it after a while. But when I was doing it like I didn' t, yeah, it was fine. It just… just when it started to get slow, I stopped using it as much.

[Participant 3, 20% completion rate]

Some participants felt the app was not beneficial or useful to them and experienced some questions as irrelevant to their situation. These factors affected the initial motivation to participate in the study, eventually leading to disengagement.

I had no problem in doing it. It was easy to use and everything but… It didn' t like add any value to me […] it felt like a task I needed to do, like another thing on my list […] when I did first I was good, like at the start […] but then I just, like, felt like something like, I don' t know, like I had to do, like, hanging over my head. I' ve gotten to a point where […] I had control of things and so I don' t really see the benefit of like doing it.

[Participant 16, 15% completion rate]

Some participants found integrating the app into their daily lives challenging, especially when the timing of notifications became inconvenient as work or education schedules changed. Even with strong initial motivation, other life priorities impacted app usage. Participants reported forgetting about the assessment despite receiving the notifications or skipping assessments due to being tired at the end of a long day.

[…] because I have like a nine to five and […] I just get distracted with life and that. And then I' ll just forget. And I' m like, “ oh, shoot!”. Like you know, I haven' t done it.

[Participant 4, 10% completion rate]

#### Improving Users’ Experience and Engagement in Smartphone-Based Research

In addition to reducing the frequency from daily to 1 or 2 times per week, participants suggested shortening the length of the smartphone-based assessments and incorporating *randomization* of the questions to ameliorate repetition and boredom. Furthermore, several participants suggested adding a section for open-ended questions, functioning as a sort of personal journal to encourage self-expression.

[…] maybe kind of like a little journal, sort of like little journal entries for each day. Maybe that would also be an interesting, interesting to look at kind of like how the day was structured […] if there were any like certain cues which can you know trigger um potentially another episode […]

[Participant 12, 87% completion rate]

Often, the changes proposed by participants moved in the direction of making the app more personalized or relevant to the user. For example, participants suggested adding information about psychosis, background music, gamification, and peer support. Another popular suggestion was the inclusion of personalized charts allowing participants to track their own changes over time, encouraging self-monitoring and self-insight.

It would be interesting to see some sort of like charting or whatnot to see like how… If your answer has been consistent or if you' ve been trending towards a certain mood or that kind of thing.

[Participant 11, 24% completion rate]

In total, 85% of the interviewed participants reported using other apps or devices to track their physical health. They particularly valued the possibility of monitoring specific aspects such as steps, body weight, menstrual cycle, or water intake. Participants were motivated to use these apps because they enabled the setting of personal goals and sometimes provided tailored content. Overall, participants appreciated the low burden of these apps: monitoring was often automated, providing personalized information without the need for regular interaction with the app.

I use like the Apple Fitness app and I use a Fitbit app.[…] I tried to do 10 K days so I measure that with my apps and yeah, just mainly my fitness, trying to like stay on top of that cause I go to the gym as well. It keep… it shows my progress and it keeps me like focused on the goal and my targets and everything.

[Participant 17, 0.8% completion rate]

#### Understanding Participants’ Attitude Toward Digital Technologies in Clinical Care

Most of the interviewed participants (80%) would be willing to use a smartphone app as a part of their day-to-day clinical care. Although they generally felt apps could be useful for encouraging self-reflection and supporting recovery, participants would like to have some level of control over the data being shared. This includes, for example, being able to decide which data are collected, who has access to them and for how long.

I would like to be asked when… Like for example like the Care coordinator would say ‘ Oh, can I check this results’ and then I would give rather than […] the ability for the care coordinator to see everything at any single time. I would prefer to be like asked like on that day the Care coordinator ‘ can I check your mental Health app, is that OK’ and me giving consent for that particular time because consent can change, you know, over time, so maybe I can be happy to share at that time and not the other time. So, I' d like that kind of feature.

[Participant 7, 32% completion rate]

Despite this generally positive attitude, participants raised some concerns on data privacy, data security, and confidentiality. Participants seemed to generally trust the safety of their clinical data as handled by the clinical team or in research settings and trusted that appropriate data protection regulations are in place. Nevertheless, privacy seems to be a greater concern when it comes to mental health compared to physical health data:

[…] you do have to be careful in certain aspects, you know on what you put out there, […] I mean it depends on what parts of the health we are talking about, some things that can be a bit sensitive, but for some things like how I was saying, for example the walking or the heart rates and stuff like that… I think it is, it is quite helpful. […] generally speaking […] if I had like a severe condition or something that I wouldn' t want to disclose so much, I would- I wouldn' t be as comfortable doing it via an app because as much as you talk about data privacy and stuff like that, there’s… there’s still, you know you' re still your information is held somewhere and you know you don' t know. Like what’s… Yeah, what could happen or how it could be used, you know?

[Participant 10, 25% completion rate]

## Discussion

### Impact of Demographic and Baseline Clinical Characteristics on Smartphone-Based Assessment Completion Rates

By integrating quantitative and qualitative data, our study helps fill the current gap in empirical evidence regarding the demographic and clinical factors that influence long-term engagement, including facilitators and barriers, in people with a FEP.

The average smartphone-based assessment completion rate was 14.5% (SD 15.96), approximately half of the encouraged use and lower than what was reported in previous studies [11,17,33]. Our lower compliance rate may be explained by the study design and duration (1 daily assessment over 12 months), which was significantly longer than the median duration of similar studies (7 days) [17]. Furthermore, to test the app under conditions that closely resemble real-world scenarios, we adopted broad inclusion criteria and did not exclude participants with more severe symptoms, as long as they were able to consent and willing to participate. Finally, participants in our study did not receive financial compensation for completing the smartphone assessments, unlike in other studies where monetary incentives may have influenced engagement. In contrast with previous literature [14,17], we did not find associations between demographic characteristics and completion rates. This might be due to the fact that, while age is thought to be a significant predictor of engagement [14], the vast majority of our participants were young adults (mean age 25.6, SD 5.3). On the other hand, after adjusting for potential confounders, completion rates were positively associated with baseline level of functioning and negatively associated with the general symptoms subscale as indexed by the PANSS and depressive symptoms as indexed by the HAM-D, consistent with previous findings [14,17]. This suggests that patients with more severe symptoms may find it challenging to engage with smartphone apps [34], resulting in inconsistent or incomplete information being collected. In these individuals, the use of financial incentives and more regular contact with researchers may help improve retention. This aspect of our findings also raises the possibility that changes in app usage could serve as a useful indicator of a patient’s well-being. For instance, in a clinical setting, a significant drop in app usage could signal the need for a mental health team to review the patient’s progression.

### Acceptability, Barriers, and Motivators That May Influence Long-Term User Engagement and Compliance With the Study App

The study app was generally found to be user-friendly and easy to use, and the repetitive nature of the assessment was praised as a positive feature, helping users become familiar with the app and making the process more efficient. On the other hand, many participants found the daily repeated questions monotonous, which contributed to reduced usage despite the automatic daily reminder. Furthermore, some participants felt the app lacked clear benefits and perceived some questions as irrelevant, whilst others found it challenging to remain engaged over a long period of time due to competing life priorities.

The majority of interviewed participants suggested that using the app once or twice a week would be ideal; this is well aligned with our quantitative results indicating an average completion rate of 14.5% (equivalent to 53 assessments in a year or once a week). Our findings also suggest that people value the low burden of passive monitoring (eg, step counts), although they are happy to actively interact with apps if there is a clear personal benefit. Most people reported indirect benefit from self-monitoring even though the app was not designed and was not intended to provide any form of mental health support. By asking participants to report on their activities and well-being, the app appeared to encourage them to regularly reflect on their experiences, possibly improving self-awareness. As suggested by several interviewed participants, monitoring apps might be beneficial, particularly if equipped with tracking features and personalized charts, allowing participants to self-monitor changes over time. To enhance motivation and sustained usage, the app should have a balanced cost-benefit ratio and incorporate regular smartphone-based assessments (once to twice a week), passive and self-monitoring tools (eg, charts), and individualized features like the possibility of setting personal goals.

Although infrequent, some participants experienced technical issues, such as the app running slowly or failing to send daily notifications. The absence of clear technical support in these circumstances highlights a missed opportunity for the research team to leverage the positive relationship with participants to monitor app usage, address these issues, and potentially improve retention. If similar apps were implemented in a clinical setting, the clinician could provide app coaching and ongoing support during regular reviews. This approach might lead to better adherence in a clinical context compared to a research setting.

The opportunity to contribute to research, help others who might experience psychosis in the future, and give back for the support received were identified as strong motivators. In line with findings from a 2024 report commissioned by the Medicines and Healthcare products Regulatory Agency and the National Institute for Health and Care Excellence [35], the importance of connection and social presence [36] emerged from several interviews. Participants enjoyed the 4-month assessments with the researcher because these provided a space to reflect and share their experiences. Human connection and support are important factors to enhance adherence to any treatment [36]. Digital psychiatry should therefore complement traditional care, serving as an additional tool rather than replacing the therapeutic relationship between patients and clinicians.

### Participants’ Attitude Toward the Implementation of Digital Technologies in Clinical Care Settings

Similarly to previous studies [6], the vast majority of participants (80%) were in favor of using a smartphone app as part of their day-to-day clinical care. Privacy and data security, however, remain a concern as legislation moves slower than technology. Mental health data are often uniquely sensitive, and the use of digital technologies could pose risks to privacy and confidentiality; if such data were leaked or stolen, it could lead to harm to health and safety, as well as increased risk of discrimination [37]. Although interviewed participants expressed trust in data handling and safety both in research and clinical settings, some concerns were raised (for example, questions about who would have access to the data and under what circumstances). These concerns should be prioritized and addressed when designing and conducting research. Equally, regulations should be regularly reviewed and guidelines updated to keep pace with the implementation of new technologies in clinical practice.

### Strengths and Limitations

This study used purposive sampling, and although those who dropped out from the main study could not be interviewed, potentially limiting insight into attrition-related barriers, efforts were made to interview participants with varying engagement, including participants with very low completion rates (0.8%). Unlike studies of short-term app use, our project evaluated experiences over a 1-year period, offering novel insights into the feasibility of long-term app use. Additionally, the combination of deductive and inductive analysis enabled the emergence of unexpected components, particularly the perceived benefit of the app on mental health and the appreciation for the relationship with researchers and the 4-month study appointments.

With regard to limitations, participants were young individuals (25 years old on average) already comfortable with technology, and therefore, our results might not be generalizable to older populations. This limitation, however, is mitigated by the fact that the Social Mind app was designed for use in people with FEP who tend to be young adults.

Participants were instructed to complete as many smartphone assessments as possible, at least twice a week. This instruction might limit the generalizability of our findings to other studies using more frequent assessments designed to capture psychological mechanisms that unfold within shorter time frames. Nonetheless, our research team considered this frequency to be a reasonable compromise between the need for regular data collection and minimizing participants’ burden over the course of a 1-year study. Finally, for the qualitative study, we only recruited from a sub-sample of participants from 2 recruiting sites in London (South London and Maudsley and Central and North West London NHS Foundation Trusts). The between-group analysis showed that participants from the qualitative sample presented with a higher level of functioning at baseline compared to the full sample. This means that the results of the qualitative component may not be fully representative of the broader population—a common issue in clinical studies with qualitative interviews in a subset of participants [38].

### Conclusions

Our study on the use of smartphone apps in people with a FEP has highlighted both barriers and motivations. Barriers included the impact of clinical symptoms, the repetitiveness of the assessments, and the difficulty of maintaining long-term engagement. However, participants also valued the repetitive nature of regular assessments, the opportunity to monitor their own progress, and perceived benefits to mental health. Overall, our findings suggest that smartphone-based research in FEP is acceptable, particularly with a frequency of 1‐2 assessments per week. Although participants expressed interest in using smartphone apps as a clinical tool, this should complement, rather than replace, the therapeutic relationship with clinicians. Patients experiencing more severe symptoms may require additional support and incentives to sustain engagement over time.

## Supplementary material

10.2196/71989 Multimedia Appendix 1 Topic guide.

10.2196/71989 Multimedia Appendix 2 Consolidated criteria for reporting qualitative studies.

10.2196/71989 Multimedia Appendix 3 Table S1 Association between baseline characteristics and smartphone-based assessment completion rates.

## Abbreviations

COREQ: Consolidated Criteria for Reporting Qualitative Research
FEP: first episode of psychosis
GAF: global assessment of functioning
GF: global functioning
HAM-D: Hamilton Rating Scale for Depression
PANSS: Positive and Negative Syndrome Scale
