# Supplementary material for: Smartphone-Based Self-Monitoring in First Episode Psychosis: Mixed-Methods Study of Barriers and Facilitators to Engagement
Source: J Med Internet Res. 2025 Aug 26;27:e71989. doi: 10.2196/71989 (PMC12380402; doi:10.2196/71989)
Supplement: Multimedia Appendix 1 [file jmir-v27-e71989-s001.doc]

**Topic-guide for qualitative interviews**

**Urban Mind app-specific questions**

1. What was your first impression of the Urban Mind app?
2. What do you like most about the app?
3. What do you like least about the app?

1. Have you experienced any technical difficulties with the app?

For example, any difficulties with the app crashing, being slow, freezing etc?

Are you receiving notifications every day?

Any issues with not knowing how to use the app or instructions being unclear?

[If yes] How did this affect your willingness to continue using the app?

1. What motivates/ed you to use the app?
2. What motivates/ed you to take part in the study?
3. What are some common reasons why you don’t complete an assessment?
4. What else could motivate you to complete the assessments?
5. If you could decide, how often would you be willing to complete the assessments?

More/less often than once a day?

1. What would you change or improve about the app?

For example, is there anything you think the app is missing that could make it more useful for you or the study?

1. Is there anything else you would like to add about your experience while taking part in the Urban Mind Social study?

**General questions**

1. Do you use any apps to track or improve your health?

What apps do you use and why?

What do you like about these apps?

What features do you use the most?

1. Do you use any devices that monitor your health such as Fitbits?
2. How do you feel about using a smartphone app or a device to track information about your health?

Do you have any concerns about this?

On a scale of 1-10, how concerned are you about this?

On a scale of 1-10, how concerned are you about data privacy?

1. Would you be willing to use an app as part of your day-to-day clinical care?

For example, would you be willing for your care coordinator to have access to your data so they can reach out to you if they felt you needed extra support?

1. Is there any data collected via a smartphone app or a device that you would not feel comfortable sharing with the clinical team?
