# Supplementary material for: Smartphone-Based Self-Monitoring in First Episode Psychosis: Mixed-Methods Study of Barriers and Facilitators to Engagement
Source: J Med Internet Res. 2025 Aug 26;27:e71989. doi: 10.2196/71989 (PMC12380402; doi:10.2196/71989)
Supplement: Multimedia Appendix 3 [file jmir-v27-e71989-s003.docx]

Table S1 Association between baseline characteristics and smartphone-assessment completion rates.

|  | **Coefficient**  **(95% CI)** | **P** | **Coefficient**  **(95% CI)** | **P** |
| --- | --- | --- | --- | --- |
|  | Unadjusted |  | Adjusted ^c^ |  |
| **PANSS Total** | -.297  (-.489, -.104) | .003^a^ | -.294  (-.492, -.096) | .004^a^ |
| **PANSS Positive** | -.769  (-1.435, -.104) | .024^a^ | -.692  (-1.384, -.001) | .050 |
| **PANSS Negative** | -.446  (-.989, .096) | .107 | -.487  (-1.051, .076) | .090 |
| **PANSS General** | -.575  (-.898, -.252) | .001^a^ | -.589  (-.923, -.254) | .001^a^ |
| **HAM_D** | -.486  (-.825, -.148) | .005^a^ | -.524  (-.874, -.174) | .004^a^ |
| **GAF disability** | .147  (.008, .286) | .038^a^ | .173  (.022, .324) | .024^a^ |
| **GAF symptoms** | .165  (.029, .302) | .018^a^ | .155  (.011, .298) | .034^a^ |
| **GF role** | 1.156  (-.309, 2.620) | .122 | 1.321  (-.385, 3.028) | .129 |
| **GF social** | 2.026  (.371, 3.680) | .017^a^ | 2.268  (.474, 4.061) | .013^a^ |
| **Age** | .260  (-.121, .642) | .181 | .275  (-.146, .697) | .200 |
| **Gender** | 1.147  (-2.896, 5.189) | .577 | 1.310  (-2.901, 5.522) | .541 |
| **Ethnicity ^b^** |  |  |  |  |
| Black | -4.195  (-9.209, .819) | .101 | -4.117  (-9.308, 1.074) | .120 |
| Asian | 1.692  (-4.229, 7.614) | .574 | 1.924  (-4.278, 8.127) | .542 |
| Other | -2.771  (-8.475, 2.932) | .340 | -2.417  (-8.353, 3.517) | . 423 |
| **Currently in Employment** | -.123  (-4.250, 4.003) | .953 | -.628  (-4.810, 3.554) | .768 |
| **Currently enrolled in education** | -1.178  (-5.774, 3.417) | .614 | .325  (-4.748, 5.398) | .900 |
| ^a^ Statistically significant associations  ^b^ White was used as reference level  ^c^ Analysis were adjusted for age, gender, ethnicity, education and occupation. | | | | |
